# The Effect of Botulinum Toxin Type A Injections on Stricture Formation, Leakage Rates, Esophageal Elongation, and Anastomotic Healing Following Primary Anastomosis in a Long- and Short-Gap Esophageal Atresia Model – A Protocol for a Randomized, Controlled, Blinded Trial in Pigs

**DOI:** 10.29337/ijsp.156

**Published:** 2021-08-11

**Authors:** Emma Svensson, Peter Zvara, Niels Qvist, Lars Hagander, Sören Möller, Lars Rasmussen, Henrik Daa Schrøder, Eva Kildall Hejbøl, Niels Bjørn, Súsanna Petersen, Kristine Cederstrøm Larsen, Jan Krhut, Oliver J. Muensterer, Mark Bremholm Ellebæk

**Affiliations:** 1 Pediatric surgery, Department of Clinical Sciences Lund, Faculty of Medicine, Lund University. Skane University Hospital Lund, 221 84 Lund, Sweden; 2 Research Unit for Urology, Department of Clinical Research, University of Southern Denmark, J. B. Winsløws Vej 4, 5000 Odense C, Denmark; 3 Research Unit for Surgery, Odense University Hospital, University of Southern Denmark, J. B. Winsløws Vej 4, 5000 Odense C, Denmark; 4 OPEN – Open Patient data Explorative Network, Odense University Hospital; Department of Clinical Research, University of Southern Denmark, J. B. Winsløws Vej 9A, 5000 Odense C, Denmark; 5 Department of Pathology, Odense University Hospital, University of Southern Denmark, J.B. Winsløws Vej 15, 5000 Odense, Denmark; 6 Department of Surgical Studies, Medical Faculty, Ostrava University, Syllabova 19, 703 00, Ostrava, Czech Republic; 7 Department of Urology, University Hospital, 17.listopadu 1790, 708 52 Ostrava, Czech Republic; 8 Department of Pediatric Surgery, Dr. von Hauner Children’s Hospital of the Ludwig-Maximilians-University Munich, Lindwurmstraße 4, 80337 Munich, Germany

**Keywords:** oesophageal atresia, botulinum toxin, anastomotic stricture

## Abstract

**Background:**

Esophageal atresia (EA) is a congenital malformation affecting 1:3000-4500 newborns. Approximately 15% have a long-gap EA (LGEA), in which case a primary anastomosis is often impossible to achieve. To create continuity of the esophagus patients instead have to undergo lengthening procedures or organ interpositions; methods associated with high morbidity and poor functional outcomes. Esophageal injections of Botulinum Toxin Type A (BTX-A) could enable primary anastomosis and mitigate stricture formation through decreased tissue tension.

**Methods and Analysis:**

In this randomized controlled blinded animal trial, 24 pigs are divided into a long- or short-gap EA group (LGEA and SGEA, respectively) and randomized to receive BTX-A or isotonic saline injections. In the LGEA group, injections are given endoscopically in the esophageal musculature. After seven days, a 3 cm esophageal resection and primary anastomosis is performed. In the SGEA group, a 1 cm esophageal resection and primary anastomosis is performed, followed by intraoperative injections of BTX-A or isotonic saline. After 14 days, stricture formation, presence of leakage, and esophageal compliance is assessed using endoscopic and manometric techniques, and in vivo and ex vivo contrast radiography. Tissue elongation is evaluated in a stretch-tension test, and the esophagus is assessed histologically to evaluate anastomotic healing.

**Ethics and Dissemination:**

The study complies with the ARRIVE guidelines for animal studies and has been approved by the Danish Animal Experimentation Council. Results will be published in peer-reviewed journals and presented at national and international conferences.

**Highlights:**

- The optimal management of long-gap esophageal atresia remains controversial
- Primary anastomosis could improve functional outcomes and reduce complications
- Botulinum Toxin Type A decreases tissue tension and could facilitate anastomosis
- Reduced tension could further abate the risk for anastomotic stricture and leakage
- We present a model to evaluate the method in long- and short-gap esophageal atresia

## 1. Introduction

### 1.1. Background

Esophageal atresia (EA) is a congenital malformation affecting 1:3000-4500 newborns [1,2]. It arises from an abnormal development of the embryonic foregut, resulting in an incomplete fusion of the upper and lower esophageal pouches [2,3]. Patients with EA require advanced surgical care during the neonatal period, and the condition contributes significantly to morbidity through infancy and childhood [4,5]. In most patients, a primary anastomosis between the esophageal pouches is feasible [6,7]. However, in 15% a long-gap EA (LGEA) is present, defined as a distance between the two pouches exceeding 2.5-3 cm or more than 3–4 vertebral bodies [8]. In these cases, a primary anastomosis is often impossible to achieve due to the high tension in the tissue. If performed, it significantly increases the risk for stricture formation and anastomotic leakage [9,10]. Current techniques to create continuity of the esophagus, including organ interposition, traction procedures, or awaiting spontaneous growth of the pouches, are associated with a high rate of postoperative complications and poor long-term functional results [11,12,13,14]. New treatment modalities facilitating primary anastomosis for patients with LGEA are therefore highly warranted.

Botulinum Toxin Type A (BTX-A) is a muscle-relaxing toxin derived from the gram-positive bacteria Clostridium botulinum. It blocks the acetylcholine release in neuromuscular junctions by cleaving the t-SNARE’s, which hinders vesicles containing acetylcholine to fuse with the presynaptic membrane and ultimately causes muscle relaxation [15,16,17]. In addition, BTX-A inhibits the release of other neurotransmitters important for tonic muscular status, including adenosine triphosphate. Previous studies from our research group have shown that injections of BTX-A into the esophageal musculature in pigs and rats lead to a significant increase in esophageal elongation [18,19]. In addition, early evidence suggests a reduced stricture formation following esophageal anastomosis [19]. We have also shown that administration of BTX-A via endoscopic techniques is feasible, and that the effect of BTX-A on esophageal elongation is dosage- and time-dependent [20,21].

While these results suggest that BTX-A injected into the esophageal musculature prior to surgery could enable primary anastomosis in cases of LGEA, it has not yet been studied in longer-term survival studies of larger animals. Furthermore, it is not known if the tissue-relaxing effect of BTX-A could reduce anastomotic stricture formation and leakage rates: two common and potentially severe complications in cases of both LGEA and short-gap EA (SGEA) [22,23]. Lastly, it is not known if an increased interval from injection to surgery affects esophageal elongation, and whether the BTX-A treatment has any detrimental or beneficial effect on anastomotic healing.

### 1.2. Hypothesis

Animals receiving injections of BTX-A into the esophageal musculature will have a lower degree of stricture formation, lower rate of anastomotic leakage, greater esophageal elongation and compliance, and more organized and complete regeneration of muscle structures with less collagen formation at the anastomosis site 14 days after esophageal resection and anastomosis, compared to the control group receiving the equivalent amount of isotonic saline.

### 1.3. Research questions

Does BTX-A, injected into the esophageal musculature pre- or intraoperatively to esophageal resection and anastomosis in an LGEA or SGEA animal model, affect anastomotic stricture formation, leakage rates, esophageal elongation and compliance, and anastomotic healing?

## 2. Methods and Analysis

### 2.1. Study set-up and location

The study will be conducted at Odense University Hospital, as part of a European collaboration between Odense University Hospital (Denmark), Ludwig-Maximilians-University Munich (Germany) and Lund University (Sweden).

### 2.2. Study design

**Figure 1:**
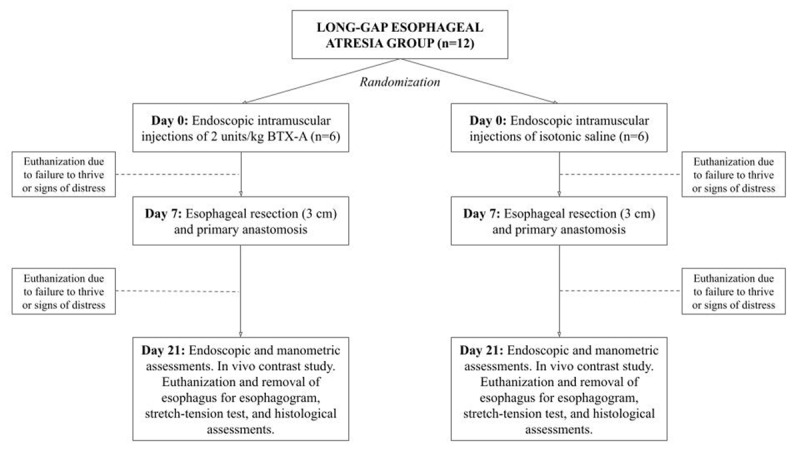
Overview of the study design, long-gap esophageal atresia group.

**Figure 2:**
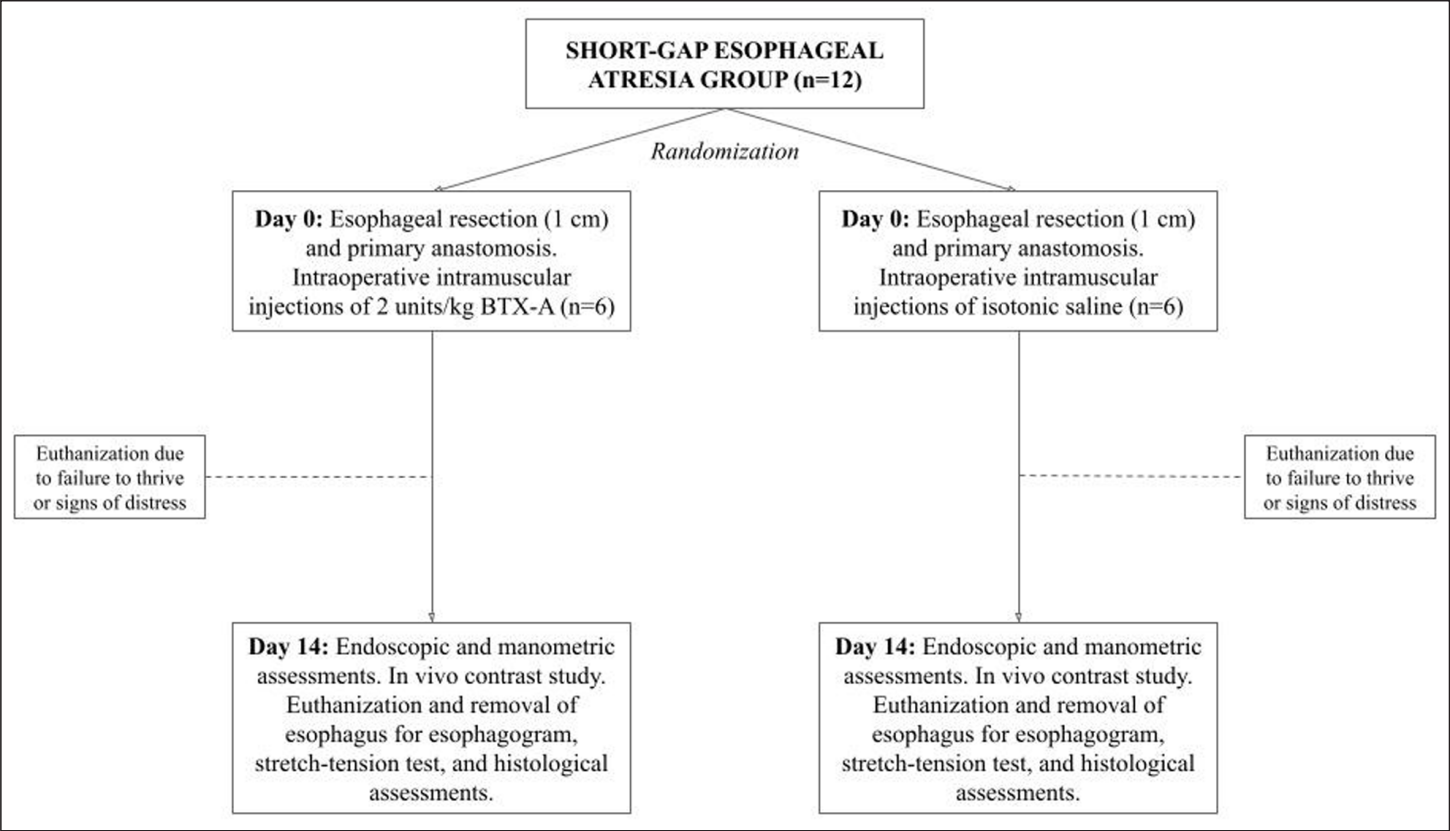
Overview of the study design, short-gap esophageal atresia group

This is an investigator-blinded randomized controlled animal trial. A total of 24 7–8-week-old Danish landrace pigs are included and divided into a LGEA or SGEA group (n = 12, respectively). Within each group, animals are randomized to receive BTX-A or 0,9% isotonic saline injections. In the LGEA group, injections are given endoscopically seven days prior to esophageal resection and anastomosis. For comparability to the clinical settings, animals in the SGEA group are given injections of BTX-A or 0,9% isotonic saline intraoperative to esophageal resection and anastomosis. At postoperative day 14, all animals are re-anesthetized and assessed to examine stricture formation, presence of anastomotic leakage, esophageal elongation and compliance, and anastomotic healing. The study design is outlined in detail in ***Figures 1*** and ***2***.

### 2.3. Sample size estimation

A previous study in rabbits found the mean Esophageal Anastomotic Stricture Index (EASI) in the intervention group to be 0.446 and mean EASI in the placebo group to be 0.303 [24]. With a standard deviation of 0.1, a significance level (alpha) of 0.05 and a power of 80%, a total of 9 animals must be included in each group. We plan to include 24 pigs in total, resulting in a power of 92% comparing the two treatment modalities between the total groups, hence allowing for possible drop-out of 10-15% while ensuring a power of at least 80%. Moreover, including 24 animals will result in a power of 61% for sub-comparisons inside the LGEA and SGEA groups.

### 2.4. Anesthesia, postoperative management and euthanasia

After a fasting period of 12 hours, pigs are premedicated with an intramuscular injection of Medetomidine 0,03 mg/kg, Midazolam 0,25 mg/kg, Ketamine 5 mg/kg and Butorphanol 0,2 mg/kg. General anesthesia is induced with Propofol 10 mg/kg. The pigs are intubated, and general anesthesia is maintained by an infusion of Propofol 15 mg/kg/h and Fentanyl 50 µg/kg/h. Antibiotic treatment with Metronidazole 20 mg/kg and Amoxicillin 10 mg/kg is given preoperatively and for three and six days after the endoscopic and surgical procedure, respectively. At the end of the surgical procedure, Xylocaine 10 mg/kg and Metamizole 25 mg/kg is given subcutaneously and intravenously, respectively. A transdermal Fentanyl patch 2 µg/kg/h is placed and, until the patch is fully efficient, intramuscular injections of Buprenorphine 0,3 mg/kg is administrated. Meloxicam 0,4 mg/kg is given orally for three days after the procedures. In the case of additional pain, oral Metamizole 25–50 mg/kg and intramuscular Buprenorphine 0,3 mg/kg are available.

Between the procedures, animals are stabled with access to heat lamps, softened food, and water. Body temperature and weight is recorded daily. All animals are assessed to evaluate the general condition, food intake, and signs of pain every 6–8 hours during the first 48 hours after the procedures, and twice daily in the remaining time. Animals are euthanized while still under general anesthesia with an intravenous dose of Pentobarbital 140 mg/kg.

### 2.5. Endoscopic and surgical procedures

#### 2.5.1. LGEA group

A 5 cm segment of the mid-to-distal esophagus is marked by endoscopic tattooing (GI SPOT, Braun Scandinavia A/S, Værløse, Denmark), using a 7,8 mm endoscope (Storz, Tuttlingen, Germany). 1,2 ml of BTX-A solution (Xeomin, Merz Pharmaceuticals GmbH, Frankfurt/Main, Germany; 2 units/kg) or 0,9% isotonic saline is injected into the esophageal musculature at 2, 10 and 20 mm distally and proximally to the tattoos, with two depots at each location. After seven days the pigs are anaesthetized, and the esophagus is exposed via a right-sided thoracotomy. A 3 cm long segment between the tattoos is resected, and an end-to-end anastomosis with full thickness interrupted sutures of Monocryl 4–0 is performed.

#### 2.5.2. SGEA group

The esophagus is exposed via a right-sided thoracotomy. A 1 cm-long esophageal segment is resected, and an end-to-end anastomosis is performed as detailed above. 1,2 ml of BTX-A solution (2 units/kg), or 0,9% isotonic saline is injected intramurally at 2, 10 and 20 mm distally and proximally to the anastomosis, with two depots at each location.

### 2.6. Assessment of stricture formation and anastomosis pressure profile

At postoperative day 14, animals are re-anaesthetized and an esophagoscopy is conducted. The presence of a macroscopic stricture, dilation of the upper pouch, and food remnants is evaluated. A UniTip Catheter 9 Ch (UniSensor AG, Attikon, Switzerland) connected to the MMS Urodynamic system (MMS, Enschede, Netherlands) is then inserted orally, advanced into the stomach and withdrawn through the esophagus at a constant speed of 5 mm/sec to measure pressure changes at the anastomosis.

Anastomotic stricture formation is assessed using a dynamic in vivo contrast study and an ex vivo contrast esophagogram. A Foley balloon catheter Ch18 is inserted orally with the tip placed approximately 8 cm above the anastomosis. After occlusion of the proximal esophagus, 40 ml of Omnipaque® contrast medium (140 mg/ml of lohexol, GE Healthcare, Brøndby, Denmark) is injected, and X-rays are taken to visualize the esophagus in vivo. The esophagus is then removed via a thoracotomy, and the specimen is clamped proximally and distally to the anastomosis. Following cannulation and infusion of Omnipaque® contrast medium to an intraluminal pressure of 20 mmHg, lateral and anteroposterior X-rays are taken.

### 2.7. Stretch-tension test

After 25 minutes of cold ischemia, the esophagus is mounted onto a stretch-tension device (LFPlus Series Universal Test Machine, Lloyd Instruments LTD, Hampshire, UK) with a distance of 3 cm between the clamps and the anastomosis placed in the center. The specimen is subjected to a 2 N preload to compensate for length difference and is stretched at a constant rate of 30 mm/min until it tears. The location of rupture is noted.

### 2.8. Tissue preparation and histological assessments

The esophagus specimen is fixed in buffered formalin and processed for paraffin embedding. Sections, with a thickness of 2 µm, will be immunohistochemically stained on BenchMark, Ventana or Omnis, Dako Agilent, instruments. To detect inflammation, antibodies against macrophages (CD68) and lymphocytes (T and B-cell markers) are employed. Appearance and degree of fibrosis is assessed using Sirius Red staining. To assess for regeneration of muscle tissue, immunohistochemical staining for desmin is used. Furthermore, antibodies against PAX7, myoD, and myogenin are employed as additional markers of skeletal muscle regeneration. To evaluate local nerve innervation, the density of nerve terminals above and below the anastomosis is compared in immunohistochemical analysis for synaptophysin.

### 2.9. Statistical analysis

#### 2.9.1. Primary outcome

The primary outcome variable is the degree of stricture formation, defined as a narrowing at the level of the anastomosis [10]. It is evaluated on the contrast esophagogram and calculated using the EASI [25]. Depending on the normality of the measurements, evaluated by a quantile-quantile plot, either a two-sample t-test or a Mann-Whitney U-test is performed to compare EASI between the BTX-A and control group. Moreover, the analysis is repeated stratified by LGEA/SGEA group.

#### 2.9.2. Secondary outcomes

Secondary outcomes include presence of anastomotic leakage, esophageal elongation and compliance, and anastomotic healing. Anastomotic leakage is defined as an effusion of contrast on the in vivo contrast study, or upon inspection and histological assessment of the specimen. Esophageal elongation is evaluated in the stretch-tension test, in which the tensile strength to cause serosa rupture (MATS-1) and a transmural rupture (MATS-2) is measured. The transmural rupture is confirmed via a drop in the load-strain curve calculated by the software (MATS-3). Total elongation and extension from preload are derived from the load-strain curve. To compare the difference of esophageal elongation, a multiple linear regression with maximum load as a function of BTX-A dose, elongation, weight, and intraoperative saturation, blood pressure and heart rate is used. In the case of deviations from distributional assumptions, bootstrapping with 1000 repetitions is applied.

The esophageal pressure measurement, including the maximum pressure at the anastomosis, provides data reflecting changes in mechanical properties of the anastomosis and esophageal wall [26]. The measurements are compared between the intervention and control groups following the analytic strategy specified for EASI. To compare the degree of inflammation, fibrosis and muscle regeneration, histological markers will be quantified. A Mann-Whitney U-test will be used for numerical measures and, for categorical measures, a Fisher exact test will be performed to compare the difference in histological markers between the intervention and placebo group. For all analysis P-values of <0.05 will be considered statistically significant. Statistical analysis will be performed using Stata (StataCorp. 2019. Stata Statistical Software: Release 16. College Station, TX: StataCorp LLC).

## 3. Ethics and Dissemination

### 3.1. Ethical considerations

The study complies with the ARRIVE guidelines for animal studies and has been approved by the Danish Animal Experimentation Council, application number 2020-15-0201-00512. Measures will be taken to ensure that no animals suffer during the trial. If an animal shows signs of distress (inappetence, inactivity, increased heart or respiratory rate and temperature, diarrhea, drooling or vomiting, teeth grinding, hanging tail, or failure to thrive) a veterinarian will assess the animal. In the case of untreatable pain, severe infection, or failure to thrive, animals will be euthanized. Efforts have been taken to reduce the number of animals needed, and to refine the techniques used.

### 3.2. Dissemination

The research protocol as well as positive, negative, or inconclusive results will be published in scientific peer-reviewed journals. Results will be presented at national and international conferences.
